# Dandelion Chloroform Extract Promotes Glucose Uptake via the AMPK/GLUT4 Pathway in L6 Cells

**DOI:** 10.1155/2018/1709587

**Published:** 2018-11-07

**Authors:** Ping Zhao, Qian Ming, Mingrui Xiong, Guanjun Song, Li Tan, Di Tian, Jia Liu, Zhao Huang, Jingyi Ma, Jinhua Shen, Qing-Hua Liu, Xinzhou Yang

**Affiliations:** ^1^Institute for Medical Biology & Hubei Provincial Key Laboratory for Protection and Application of Special Plants in the Wuling Area of China, College of Life Sciences, South-Central University for Nationalities, Wuhan 430074, China; ^2^National Demonstration Center for Experimental Ethnopharmacology Education, South-Central University for Nationalities, Wuhan 430074, China; ^3^School of Pharmaceutical Sciences, South-Central University for Nationalities, 182 Min-Zu Road, Wuhan 430074, China

## Abstract

The number of patients with type 2 diabetes mellitus (T2DM) is increasing rapidly worldwide. Glucose transporter 4 (GLUT4) is one of the main proteins that transport blood glucose into the cells and is a target in the treatment of T2DM. In this study, we investigated the mechanism of action of dandelion chloroform extract (DCE) on glucose uptake in L6 cells. The glucose consumption of L6 cell culture supernatant was measured by a glucose uptake assay kit, and the dynamic changes of intracellular GLUT4 and calcium (Ca^2+^) levels were monitored by laser scanning confocal microscopy in L6 cell lines stably expressing IRAP-mOrange. The GLUT4 fusion with the plasma membrane (PM) was traced via myc-GLUT4-mOrange. GLUT4 expression and AMP-activated protein kinase (AMPK), protein kinase B (PKB/Akt), protein kinase C (PKC), and phosphorylation levels were determined by performing western blotting. GLUT4 mRNA expression was detected by real-time PCR. DCE up-regulated GLUT4 expression, promoted GLUT4 translocation and fusion to the membrane eventually leading to glucose uptake, and induced AMPK phosphorylation in L6 cells. The AMPK inhibitory compound C significantly inhibited DCE-induced GLUT4 expression and translocation while no inhibitory effect was observed by the phosphatidylinositol 3-kinase (PI3K) inhibitor Wortmannin and PKC inhibitor Gö6983. These data suggested that DCE promoted GLUT4 expression and transport to the membrane through the AMPK signaling pathway, thereby stimulating GLUT4 fusion with PM to enhance glucose uptake in L6 cells. DCE-induced GLUT4 translocation was also found to be Ca^2+^-independent. Together, these findings indicate that DCE could be a new hypoglycemic agent for the treatment of T2DM.

## 1. Introduction

Type 2 diabetes mellitus (T2DM) is known as non-insulin-dependent diabetes mellitus and accounts for 90%–95% of all cases of diabetes [1]. Abnormal glucose and lipid metabolism is the main feature of T2DM. In the early stages of disease, pancreatic *β*-cells retain the ability to secrete insulin but the patient develops insulin resistance, resulting in the compensatory secretion of more insulin from pancreatic *β*-cells to maintain normal blood glucose levels. When *β*-cells lose this compensatory ability, blood glucose levels rise, causing T2DM [2].

Currently available medications for the treatment of diabetes are divided into four categories: insulin, insulin secretagogues, insulin sensitizers, and prandial glucose regulators. However, many of these drugs have serious adverse effects in patients with poor tolerance. Medicinal plants are increasingly being used in modern clinical medicine and have been administered as alternative treatments for T2DM, with reported superior curative effects and few side effects [3]. In this study, we aimed to identify new hypoglycemic drugs from traditional Chinese medicine.

Glucose transporter (GLUT)4 is a member of the GLUT/SLC4A family, which is widely distributed in skeletal muscle, myocardium, adipose tissue, kidney, and the brain [4–7]. In the basal state, GLUT4 exists in the special intracellular components, such as GLUT4 storage vesicles (GSVs). The pathways of insulin-stimulated glucose transport into the cell include GSVs sorting, trafficking, and finally fusion with the PM [8]. Current research suggests that GLUT4 transmembrane transport of glucose into the cell is the rate-limiting step of glucose uptake and that T2DM is associated with the loss of GLUT4 expression and translocation [9, 10]. Therefore, GLUT4 is considered to be a potential target for the treatment of T2DM.

Dandelion (*Taraxacum mongolicum *Hand.-Mazz.) is a herbaceous perennial plant belonging to Asteraceae family. Previous study exhibited that it has antioxidant [11], antitumor [12], and anti-inflammatory [13] properties. Moreover, aqueous extract of dandelion was also reported to improve lipid metabolism in T2DM rats [14]. However, the effect of DCE on the expression and translocation of GLUT4 in the treatment of T2DM is unexplored. In the present study, we found that DCE promoted GLUT4 translocation to the plasma membrane (PM), resulting in enhanced glucose uptake in L6 cells.

## 2. Materials and Methods

### 2.1. Reagents and Solutions

Alpha MEM (*α*-MEM) medium and penicillin–streptomycin solution were purchased from Gibco (Gran Island, NY, USA). Fetal bovine serum (FBS) was purchased from Hyclone (Logan, UT, USA). The glucose uptake assay kit was purchased from Cayman Chemical Company (Ann Arbor, MI, USA). Fluo-4 AM was purchased from Invitrogen (Camarillo, CA, USA). Primary antibodies against GLUT4, AMPK, and p-AMPK were purchased from Cell Signaling Technology (Beverly, MA, USA). Wortmannin and BAPTA-AM were purchased from Sigma (St. Louis, MO, USA). Compound C was purchased from Selleckchem (Houston, TX, USA). Gö6983 was purchased from EMD Millipore (Billerica, MA, USA). The physiological salt solution (PSS) contained the following composition (mM): 135 NaCl, 5 KCl, 1 MgCl_2_, 2 CaCl_2_, 10 HEPES, and 10 glucose (pH 7.4); extracellular Ca^2+^-free PSS (mM): 135 NaCl, 5 KCl, 1 MgCl_2_, 0.5 EGTA, 10 HEPES, and 10 glucose (pH 7.4)

### 2.2. Dandelion Extraction

Dandelion plants were collected from Shanxi Province, China, and were authenticated by Professor Ding-Rong Wan, School of Pharmaceutical Sciences, South-Central University for Nationalities (Wuhan, China). A voucher specimen has been deposited as a voucher specimen in the South-Central University for Nationalities herbarium.

Dried dandelion was ground into powder. The components were extracted by refluxing with 75% ethanol solution three times and centrifuging at 4800× g for 15 minutes. Next, the supernatant was collected, evaporated to dryness on a rotary evaporator to obtain the ethanol extract, thereafter dissolved in ultrapure water, and extracted three times with petroleum ether to remove the oil. Afterwards, the supernatant was extracted three times with chloroform and further dried with a rotary evaporator to obtain the DCE. DCE was dissolved in 3% dimethyl sulfoxide and used for bioactivity assay.

### 2.3. Cell Line Construction and Cell Culture

Insulin-responsive aminopeptidase (IRAP) is a protein that strongly colocalizes with GLUT4 [15, 16] and can be used as a reporter molecule to reflect the translocation of GLUT4 [17, 18]. Stable expression of IRAP in the L6 cell line (L6 IRAP-mOrange) was determined as we previously described [19]. L6 cells were cultured in *α*-MEM with 10% FBS and supplemented with penicillin (100 U/mL) and streptomycin (100 *μ*g/mL) at 37°C in a 5% CO_2_ environment.

### 2.4. Glucose Uptake Assay

L6 cells were seeded into 96-well culture plates and cultured until 100% confluence. Next, cells were starved for 2 hours with serum-free *α*-MEM medium and then treated with DCE or 100 nM insulin or vehicle control dissolved in 100 *μ*L serum-free *α*-MEM medium containing 150 *μ*g/mL glucose uptake and transport probe 2-NBDG. Plates were incubated at 37°C with 5% CO_2_ for 30 minutes. Following the treatment, the glucose uptake by cells was detected using an Infinite M200 Pro microplate reader (Tecan, Groedig, Austria) at 485 nm as the excitation wavelength and at 535 nm as the emission wavelength. Three independent experiments were conducted, comparing the control group (control), the insulin group (insulin), and the added drug group (DCE).

### 2.5. Monitoring of GLUT4 Translocation

L6 IRAP-mOrange cells were grown on glass coverslips at 37°C overnight and then starved in serum-free *α*-MEM for 2 hours. The dynamic changes of IRAP-mOrange translocation were observed using the LSM700 laser scanning confocal microscope (Carl Zeiss, Jena, Germany). Changes in IRAP-mOrange fluorescence intensity on the cell membrane were recorded before and after treatment with DCE to reflect the GLUT4 translocation.

### 2.6. Assay of GLUT4 Fusion with PM

The three gene fragments of myc, GLUT4, and mOrange were inserted into the GV348 plasmid to obtain the GV348-myc-GLUT4-mOrange plasmid and transfect it into L6 cells by lentivirus. GLUT4myc cDNA was constructed by inserting the human c-myc epitope (14 amino acids) into the first ectodomain of GLUT4, as described in [20]. A single clone containing the highest fluorescence intensity was selected and cultured for the following experiments. The GV348-myc-GLUT4-mOrange L6 cells on circular glass slides were starved with serum-free medium for 2 hours and then treated with 30 *μ*g/mL DCE or 100 nM insulin for 30 minutes. Next, the cells were fixed with 3% paraformaldehyde, blocked in 2% (w/v) BSA in PBS, and incubated with mouse anti-myc antibody (1:200 dilution) [19]. Cells were subsequently labeled with anti-mouse-FITC-conjugated secondary antibody (1: 200 dilution). The intensity of Green fluorescent protein (FITC) and mOrange fluorescence was measured by the LSM700 microscope (Carl Zeiss).

### 2.7. Detection of Intracellular *Ca*^2+^ Levels

L6 IRAP-mOrange cells were grown on glass coverslips at 37°C overnight and then starved in serum-free *α*-MEM for 2 hours. Cells were incubated in PSS containing 2 *μ*M Fluo-4 AM for 15 minutes to stain intracellular Ca^2+^ and then superfused with dye-free PSS for 10 minutes [21]. Afterwards, the fluorescence intensity of Fluo-4 AM was observed using the LSM700 microscope (Carl Zeiss).

### 2.8. Western Blotting

Cells were starved with serum-free medium for 2 hours and then treated with Wortmannin (100 nM for 30 minutes), Gö6983 (10 *μ*M for 30 minutes), compound C (10 *μ*M for 30 minutes), DCE (30 *μ*g/mL for 5 minutes or 30 minutes), insulin (100 nM for 30 minutes), phorbol ester (PMA, 200 nM for 4 hours), or metformin (100 *μ*g/mL overnight). Next, the cells were placed on ice, washed three times with cold PBS, and treated with a protease inhibitor cocktail (Roche, Basel, Switzerland) and phosphatase inhibitor cocktail (Selleckchem, USA) at 4°C. Cells were then lysed as described previously [19]. Equal amounts of protein were separated by 10% (v/v) SDS-PAGE and transferred onto a polyvinylidene difluoride (PVDF) membrane. The membranes were incubated overnight at 4°C with primary antibodies and then with corresponding horseradish peroxidase-conjugated secondary antibodies for 1 hour. The intensity of protein bands was quantitated using a ChemiDoc XRS system (Bio-Rad, CA, USA).

### 2.9. RNA Isolation and Quantitative Real-Time PCR

L6 cells were starved with serum-free medium for 2 hours and pretreated with different concentrations of drugs for 30 minutes. Total RNA was extracted using TRIzol reagent (Invitrogen) including chloroform extraction and isopropanol precipitation. The RNA initial extract of each sample was washed and dried with 75% ethanol and then dissolved in 50 *μ*L DEPC water. The quality of each RNA extraction was confirmed by electrophoresis on a 1% agarose gel. Next, 2 *μ*g of total RNA from each sample was reverse-transcribed using the RevertAid First Strand cDNA Synthesis Kit (Thermo Scientific, USA) in a 20 *μ*L reaction according to the manufacturer's protocol. cDNA products were diluted with RNase-free water, then real-time PCR was performed using the FastStart Universal SYBR Green PCR Master ROX (Roche) system on the 7500 Fast Real-Time PCR System instrument (Applied Biosystems). Primer sequences were as follows: rat Gapdh (NCBI RefSeq NM_017008.4), F: 5′-TACAGCAACAGGGTGGTGGAC-3′, R: 5′-GGGATGGAATTGTGAGGGAGA-3′; rat GLUT4 (NCBI RefSeq NM_012751.1), F: 5′-CTTCCTTCTATTTGCCGTCCTC-3′, R: 5′-GCTGCTGTTTCCTTCATCCTG-3′. Relative quantification results obtained for the target genes were normalized using the 2^-ΔΔCT^ method.

### 2.10. Data Analysis

Data were expressed as the mean ± SEM. Two groups of data were compared by the Student's t-test. Values of* P *< 0.05 were considered statistically significant.

## 3. Results

### 3.1. DCE Increased Glucose Uptake in L6 Cells

**Figure 1:**
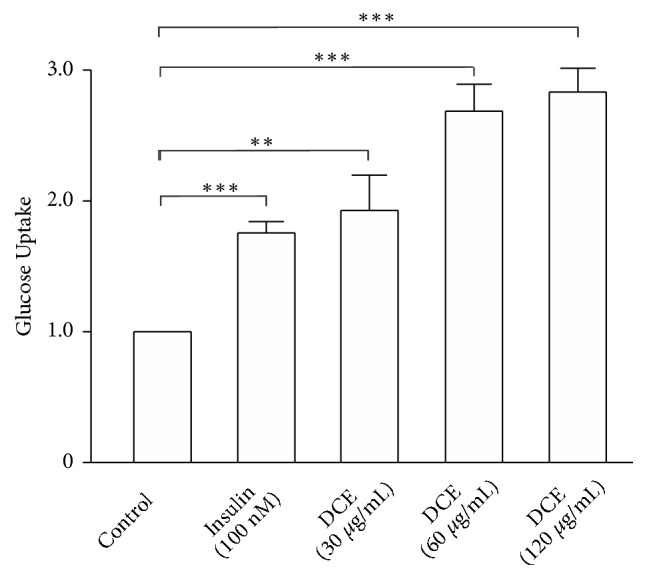
DCE increased glucose uptake in L6 cells. Compared with the control group, the glucose uptake of L6 cells was significantly increased in a dose-dependent manner following treatment with 30 *μ*g/mL, 60 *μ*g/mL, and 120 *μ*g/mL of DCE for 30 minutes. *∗∗P *< 0.01 and *∗∗∗ P *< 0.001.

The effect of DCE on glucose uptake in L6 cells was investigated, followed by glucose uptake assay kit. We found that 30, 60, and 120 *μ*g/mL of DCE significantly increased glucose uptake in L6 cells (Figure 1) in a dose-dependent manner.

### 3.2. DCE Promoted GLUT4 Translocation to the PM of L6 Cells

**Figure 2:**
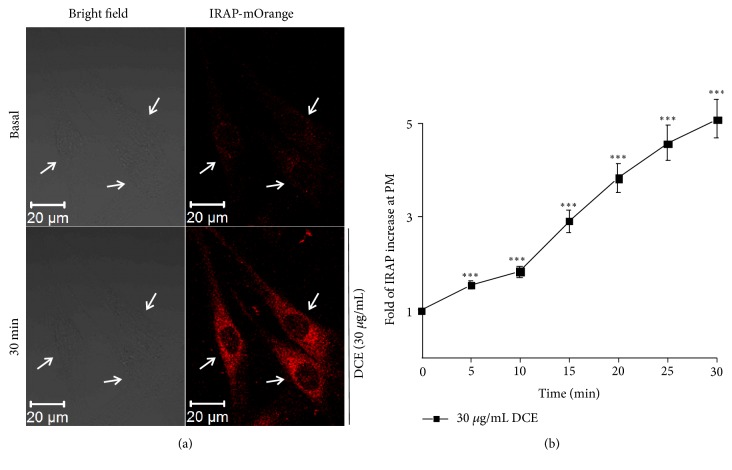
DCE promoted GLUT4 translocation in L6 cells. (a) The bright field image on the left shows the basic morphology of L6 IRAP-mOrange cells at basal level and 30 minutes after treatment with 30 *μ*g/mL DCE. The images on the right show the significant enhancement of L6 IRAP-mOrange fluorescence intensity after the addition of 30 *μ*g/mL DCE for 30 minutes. (b) Immediately after the addition of 30 *μ*g/mL of DCE until 30 minutes later, the fluorescence intensity of the L6 IRAP-mOrange cell membrane was significantly enhanced. *∗∗∗P *< 0.001.

To explore the mechanism of the DCE-induced increase in glucose uptake by L6 cells, we measured the IRAP-mOrange fluorescence intensity of the L6 IRAP-mOrange cell membrane by laser confocal microscopy. We found that 30 *μ*g/mL of DCE significantly enhanced the IRAP-mOrange fluorescence intensity (Figure 2(a)) and also significantly increased IRAP expression on the cell membrane (Figure 2(b)). Because of the known strong colocalization of IRAP and GLUT4 [15, 22], these data imply that DCE promotes GLUT4 translocation to the PM. Therefore, we hypothesized that DCE also increases the expression of GLUT4.

### 3.3. DCE Increased the Expression of GLUT4 in L6 Cells

**Figure 3:**
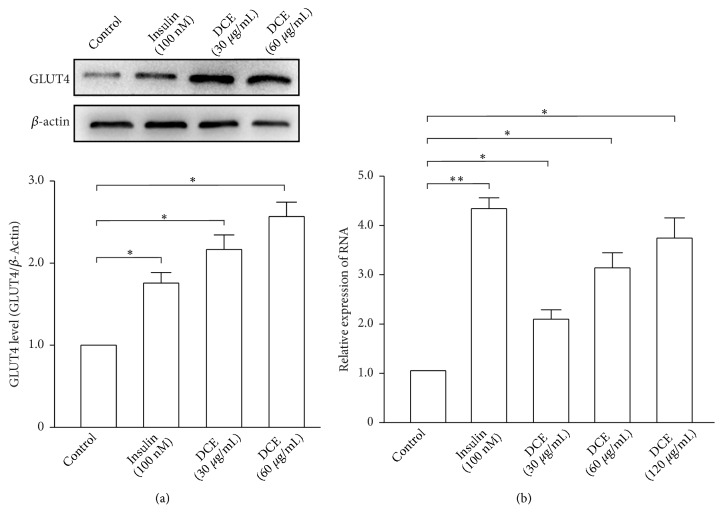
DCE increased the expression of GLUT4 in L6 cells. (a) The GLUT4 protein level of L6 cells was significantly increased after the addition of 30 *μ*g/mL and 60 *μ*g/mL DCE for 30 minutes. Summary results are from three independent experiments. *∗P *< 0.05. (b) 100 nM insulin and different concentrations of DCE induced a significant increase in* GLUT4* mRNA expression of L6 cells compared with the control in 30 minutes. The results of three independent experiments were statistically analyzed. *∗P *< 0.05 and *∗∗P *< 0.01.

To verify our hypothesis, we used western blotting and real-time PCR to detect GLUT4 protein and mRNA expression, respectively, in L6 cells. Both were found to significantly increase in a dose-dependent manner after treatment of L6 cells with different concentrations of DCE (Figures 3(a) and 3(b)). These results showed that DCE not only promoted GLUT4 translocation to the PM, but also increased the expression of GLUT4 in L6 cells.

### 3.4. DCE Promoted GLUT4 Translocation and GLUT4 Expression in L6 Cells Mainly through the AMPK Signaling Pathway

**Figure 4:**
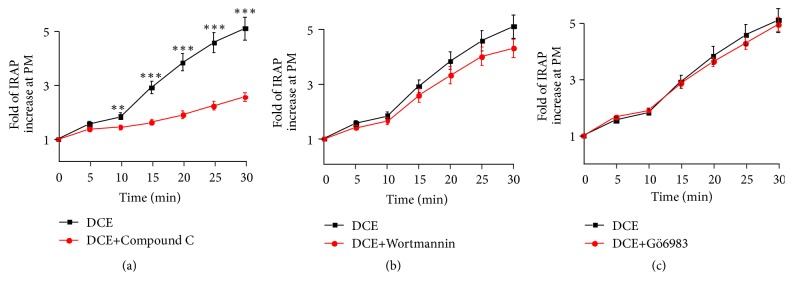
Compound C inhibited DCE-induced GLUT4 translocation. (a) The AMPK signaling pathway inhibitor compound C treated L6 cell (10 *μ*M for 30 minutes) significantly inhibited the GLUT4 translocation induced by 30 *μ*g/mL DCE. *∗∗P *< 0.01 and *∗∗∗P *< 0.001. (b) The PI3K/Akt signaling pathway inhibitor Wortmannin treated L6 cell (100 nM for 30 minutes) had no effect on 30 *μ*g/mL DCE-induced GLUT4 translocation. (c) The PKC signaling pathway inhibitor Gö6983 treated L6 cell (10 *μ*M for 30 minutes) had no effect on 30 *μ*g/mL DCE-induced GLUT4 translocation.

**Figure 5:**
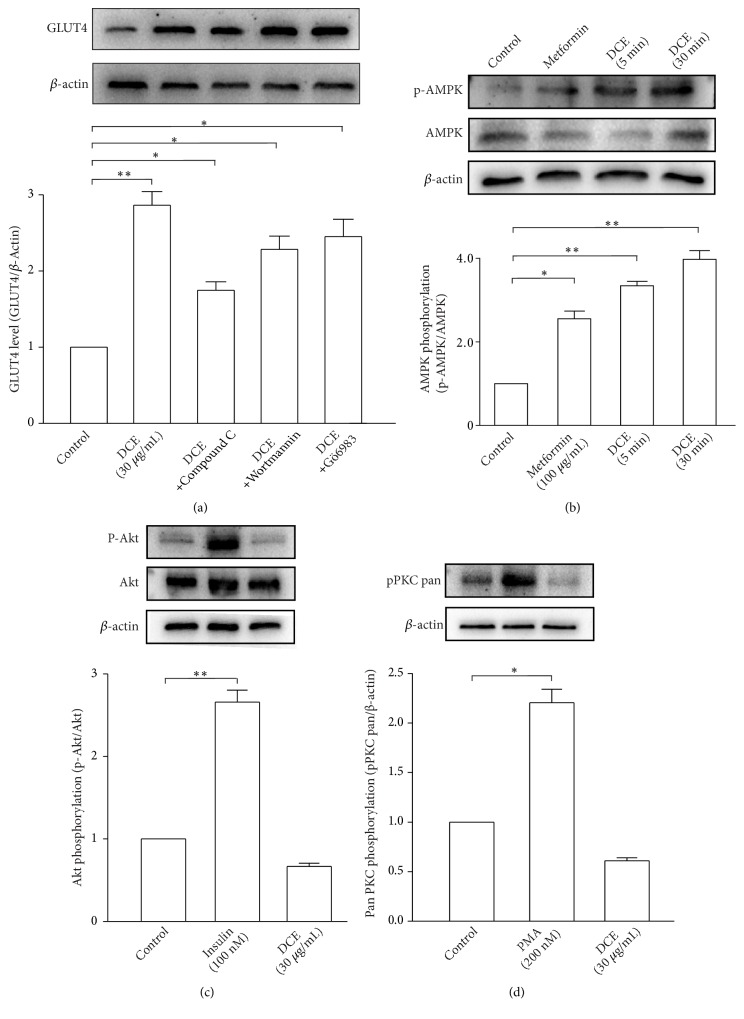
DCE increased the phosphorylation of AMPK in L6 cells. (a) The AMPK signaling pathway inhibitor compound C remarkably attenuated DCE-induced GLUT4 protein expression, but the inhibitory effects of Wortmannin and Gö6983 were less obvious. Summary results are from three experiments. *∗P *< 0.05 and *∗∗P *< 0.01. (b) AMPK phosphorylation in L6 cells was significantly increased after the addition of 30 *μ*g/mL DCE for 5 minutes and 30 minutes. Summary results are from three independent experiments. *∗P *< 0.05 and *∗∗P *< 0.01. (c) 30 *μ*g/mL DCE had no effect on Akt phosphorylation level rather than 100 nM insulin after the addition of DCE or insulin for 30 minutes, respectively. Summary results are from three independent experiments. *∗∗P *< 0.01. (d) 30 *μ*g/mL DCE had no effect on PKC phosphorylation level rather than 200 nM phorbol ester (PMA) after the addition of DCE for 30 minutes or PMA for 4 hours, respectively. Summary results are from three independent experiments. *∗P *< 0.05.

Next, we attempted to identify the signal transduction pathway(s) involved in DCE-induced GLUT4 translocation in L6 cells. L6 IRAP-mOrange cells were treated with compound C (AMPK inhibitor), Wortmannin (PI3K inhibitor), or Gö6983 (PKC inhibitor). Compound C significantly inhibited the promoting effect of DCE on GLUT4 translocation in L6 cells (Figure 4(a)), but Wortmannin and Gö6983 had no inhibitory effect (Figures 4(b) and 4(c)). Compound C also remarkably inhibited the promoting effect of DCE on GLUT4 expression in L6 cells; similarly, Wortmannin and Gö6983 had no effect (Figure 5(a)). DCE significantly increased the phosphorylation of AMPK in L6 cells (Figure 5(b)), but DCE did not cause an increase in the phosphorylation of Akt and PKC in L6 cells (Figures 5(c) and 5(d)). These findings affirm that DCE promotes GLUT4 expression and translocation mainly through the AMPK signaling pathway.

### 3.5. DCE-Induced Fusion of GLUT4 at the PM in myc-GLUT4-mOrange L6 Cells

**Figure 6:**
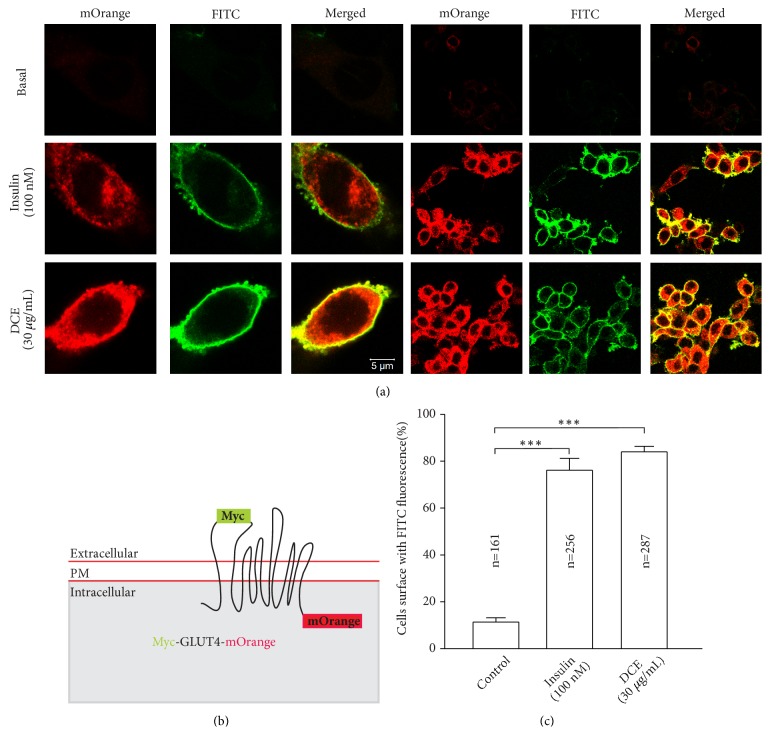
DCE increased the GLUT4 fusion with PM. (a) L6 cells were transfected with plasmid GV348-myc-GLUT4-mOrange encoding a mOrange fusion protein with myc epitope-tagged GLUT4 (myc-GLUT4-mOrange). Cells were stimulated with 100 nM insulin or 30 *μ*g/mL DCE for 30 minutes, respectively. Then fixed and hybridized with specific immunofluorescent antibodies, FITC fluorescence was measured. Scale: 5 *μ*m. (b) GLUT4 fusion protein for detection of GLUT4 translocation and PM surface exposure. (c) Statistical percentage of FITC positive cells in mOrange positive cells. Summary results are from three independent experiments. *∗∗∗P *< 0.001.

In order to further verify the mechanism of DCE-induced glucose uptake, we explored the effect of DCE on GLUT4 fusion with PM. In the absence of insulin and DCE, the anti-myc antibodies on the cell surface were not detected in the L6 cells expressing myc-GLUT4-mOrange. However, after treatment with 100 nM insulin or 30 *μ*g/mL DCE for 30 minutes, the FITC fluorescence on the cell surface was significantly increased (Figures 6(a) and 6(b)). This phenomenon indicates that DCE does increase the fusion of GLUT4 vesicles into PM to increase glucose uptake.

### 3.6. DCE-Induced GLUT4 Translocation was *Ca*^2+^-Independent

**Figure 7:**
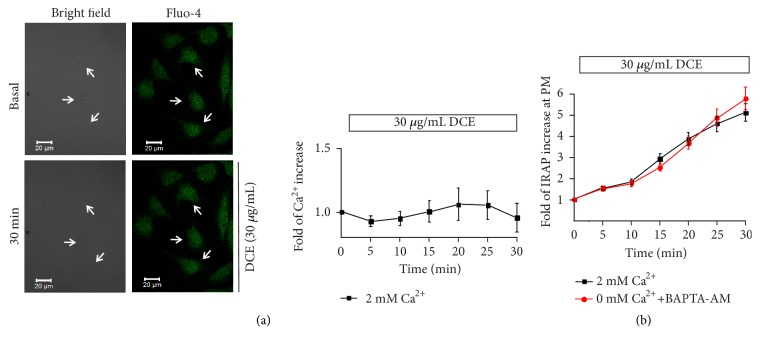
DCE-induced GLUT4 translocation was Ca^2+^-independent. (a) The confocal image on the left shows the basic morphology of L6 IRAP-mOrange cells and the fluorescence intensity of intracellular Ca^2+^ at basal level and after treatment with 30 *μ*g/mL DCE for 30 minutes, respectively. The corresponding curve on the right shows that no significant change was seen in the level of intracellular Ca^2+^ in L6 cells after the addition of 30 *μ*g/mL DCE. (b) Changes in IRAP fluorescent intensity at the PM after the addition of 30 *μ*g/mL DCE under 2 mM extracellular Ca^2+^ or 0 mM extracellular Ca^2+^ conditions with 10 *μ*M BAPTA-AM.

Previous studies have reported that Ca^2+^ plays a crucial role in insulin-induced GLUT4 translocation [23]. To determine whether the DCE-induced GLUT4 translocation is related to Ca^2+^ or not, we observed intracellular Ca^2+^ levels in L6 cells. No significant change in intracellular Ca^2+^ levels of L6 cells was detected after the addition of 30 *μ*g/mL DCE (Figure 7(a)). We next treated L6 IRAP-mOrange cells with 0 mM extracellular Ca^2+^ and the intracellular calcium chelator BAPTA-AM (10 *μ*M) and detected the fluorescence intensity of IRAP-mOrange at the PM. We found that DCE-induced GLUT4 translocation was not affected by the absence of Ca^2+^ (Figure 7(b)), suggesting that DCE-induced GLUT4 translocation is not dependent on Ca^2+^.

## 4. Discussion

In the present study, we aimed to identify a new hypoglycemic drug from traditional Chinese medicine. This study exhibited that DCE enhanced GLUT4 expression and promoted GLUT4 translocation to the PM through activating the AMPK signaling pathway, which ultimately increased glucose uptake in L6 cells in a dose-dependent manner (Figure 1). These results indicated the potentiality of DCE which could be used as a new drug for the treatment of T2DM.

Next, we investigated the mechanism of DCE that can increase glucose uptake in L6 cells. Glucose is mainly transported from the blood to skeletal muscle and adipose cells by GLUT4. Following insulin stimulation, GLUT4 translocates from GLUT4 storage vesicles (GSV) to the PM and transports glucose into the cells [24, 25]; indeed, GLUT4 transmembrane transport of glucose into the cell is the rate-limiting step of glucose uptake [4, 10]. Therefore, we hypothesized that DCE increased the glucose uptake in L6 cells by promoting the translocation of GLUT4. To verify this, we studied L6 IRAP-mOrange cells as the IRAP and GLUT4 strongly colocalize. Furthermore, IRAP could be used as a reporter molecule to reflect GLUT4 translocation into L6 cells by detecting dynamic changes in IRAP-mOrange fluorescence intensity through laser confocal microscopy [17, 18]. We found that 30 *μ*g/mL of DCE significantly increased IRAP expression on the cell membrane (Figure 2(b)), indicating that DCE promoted GLUT4 translocation to the PM. This implied that DCE increased glucose uptake in L6 cells by promoting GLUT4 translocation. We also found that 30 *μ*g/mL of DCE significantly enhanced the fluorescence intensity of IRAP-mOrange (Figure 2(a)), suggesting that DCE simultaneously increased IRAP expression, indicative of an increase in GLUT4 expression.

Western blotting and real-time PCR analyses of GLUT4 protein and mRNA expression further corroborated our findings. After treating with DCE, GLUT4 expression of L6 cells was significantly increased in a dose-dependent manner (Figures 3(a) and 3(b)). These results manifested that DCE not only promoted GLUT4 translocation, but also increased GLUT4 expression in L6 cells, leading to an increase in their glucose uptake.

Recent studies revealed that multiple signaling pathways are associated with GLUT4 translocation, including the insulin-stimulated PI3K/Akt signaling pathway [26, 27] and the antihyperglycemic metformin-induced AMPK signaling pathway [28–30]. Additionally, the PKC signaling pathway was shown to be related to GLUT4 translocation [31–33]. To determine whether these signaling pathways were involved in DCE-induced GLUT4 translocation or not, we treated L6 IRAP-mOrange cells with compound C, Wortmannin, and Gö6983. Compound C significantly inhibited DCE-induced GLUT4 translocation (Figure 4(a)), but Wortmannin and Gö6983 showed no inhibitory effect (Figures 4(b) and 4(c)). Similar results were observed at the protein level (Figure 5(a)), while DCE was also found to increase AMPK (Figure 5(b)) rather than Akt (Figure 5(c)) and PKC (Figure 5(d)) phosphorylation in L6 cells, indicating that DCE promotes GLUT4 translocation in L6 cells mainly through the AMPK signaling pathway, rather than the PI3K/Akt or PKC signaling pathways. We also observed the incomplete inhibition of DCE-induced GLUT4 translocation and expression by compound C (Figures 4(a) and 5(a)). This might have happened due to the promotion of GLUT4 translocation in L6 cells by DCE through other unknown mechanisms. However, to justify these findings, further study is needed.

A previous study revealed that the fusion of GLUT4 vesicles with PM is essential for glucose uptake [8]. In order to further verify the effect of DCE on glucose uptake, the immunofluorescence test was performed and showed that there was no obvious intracellular GLUT4 expression phenomenon and the GLUT4 vesicles-PM fusion step on the cell surface was under resting conditions. After treatment with insulin or DCE, we found that 30 *μ*g/mL DCE significantly increased the expression of intracellular GLUT4 and the gain of GLUT4 on the cell surface compared with control (Figures 6(a) and 6(b)), indicating that DCE can produce more GLUT4 and drive the GLUT4 vesicles insertion into the PM to accelerate glucose uptake. In addition, GLUT4 vesicles docked and fused with PM, and only the GLUT4 which fused with PM can transport glucose into the cells. We measured GLUT4 near the cell membrane including both GLUT4 docking to PM and GLUT4 fusing with PM. This might be one of the reasons why the fold of glucose uptake is slightly lower than the GLUT4 translocation under DCE stimulation.

Previous studies reported that Ca^2+^ plays a key role in insulin-induced GLUT4 translocation [22, 23]. To further investigate the mechanism of DCE that can promote GLUT4 translocation in L6 cells, we studied the relationship between Ca^2+^ and GLUT4 translocation and observed no significant effect of DCE on the level of intracellular Ca^2+^ in L6 cells (Figure 7(a)). Moreover, DCE-induced GLUT4 translocation remained unchanged after treatment with 0 mM extracellular Ca^2+^ and BAPTA-AM (10 mM) (Figure 7(b)). Our results showed that, unlike insulin, DCE-induced GLUT4 translocation did not depend on Ca^2+^. We speculated that the reason behind this is that the DCE does not act on the insulin signaling pathway.

In summary, DCE promoted GLUT4 translocation and expression in L6 cells through the AMPK signaling pathway and, thereby, stimulated GLUT4 fusion with PM to increase glucose uptake, while GLUT4 translocation was independent of Ca^2+^. However, a limitation of our study is that we conducted* in vitro* experiments only at the cellular level. Therefore, the hypoglycemic effect of DCE at the animal level and identification of the active ingredients of DCE require further study.

## 5. Conclusion

The present study showed that DCE promoted L6 cell GLUT4 translocation and expression through the AMPK signaling pathway, thus increasing glucose uptake by the GLUT4-PM fusion step. These results suggested the possibility of DCE being used as a novel hypoglycemic agent for the treatment of T2DM.

## Data Availability

All data generated or analyzed during this study are included within the article.

## References

[1] Guariguata L., Whiting D., Weil C., Unwin N. (2011). The International Diabetes Federation diabetes atlas methodology for estimating global and national prevalence of diabetes in adults. *Diabetes Research and Clinical Practice*.

[2] Lauritano C., Ianora A. (2016). Marine organisms with anti-diabetes properties. *Marine Drugs*.

[3] Xiong M., Huang Y., Liu Y. (2018). Antidiabetic Activity of Ergosterol from Pleurotus Ostreatus in KK-A(y) Mice with Spontaneous Type 2 Diabetes Mellitus. *Molecular Nutrition & Food Research*.

[4] Govers R. (2014). Molecular mechanisms of GLUT4 regulation in adipocytes. *Diabetes & Metabolism*.

[5] Kotani K., Peroni O. D., Minokoshi Y., Boss O., Kahn B. B. (2004). GLUT4 glucose transporter deficiency increases hepatic lipid production and peripheral lipid utilization. *The Journal of Clinical Investigation*.

[6] Zisman A., Peroni O. D., Abel E. D. (2000). Targeted disruption of the glucose transporter 4 selectively in muscle causes insulin resistance and glucose intolerance. *Nature Medicine*.

[7] Carvalho E., Kotani K., Peroni O. D., Kahn B. B. (2005). Adipose-specific overexpression of GLUT4 reverses insulin resistance and diabetes in mice lacking GLUT4 selectively in muscle. *American Journal of Physiology-Renal Physiology*.

[8] Xie X., Gong Z., Mansuy-Aubert V. (2011). C2 domain-containing phosphoprotein CDP138 regulates GLUT4 insertion into the plasma membrane. *Cell Metabolism*.

[9] Abel E. D., Peroni O., Kim J. K. (2001). Adipose-selective targeting of the GLUT4 gene impairs insulin action in muscle and liver. *Nature*.

[10] Lacombe V. A. (2014). Expression and Regulation of Facilitative Glucose Transporters in Equine Insulin-Sensitive Tissue: From Physiology to Pathology. *ISRN Veterinary Science*.

[11] Mingarro D. M., Plaza A., Galán A., Vicente J. A., Martínez M. P., Acero N. (2015). The effect of five Taraxacum species on in vitro and in vivo antioxidant and antiproliferative activity. *Food & Function*.

[12] Takasaki M., Konoshima T., Tokuda H. (1999). Anti-carcinogenic activity of Taraxacum plant. II. *Biological & Pharmaceutical Bulletin*.

[13] Jiang S.-H., Ping L.-F., Sun F.-Y., Wang X.-L., Sun Z.-J. (2016). Protective effect of taraxasterol against rheumatoid arthritis by the modulation of inflammatory responses in mice. *Experimental and Therapeutic Medicine*.

[14] Cho S.-Y., Park J.-Y., Park E.-M. (2002). Alternation of hepatic antioxidant enzyme activities and lipid profile in streptozotocin-induced diabetic rats by supplementation of dandelion water extract. *Clinica Chimica Acta*.

[15] Jiang L., Fan J., Bai L. (2008). Direct quantification of fusion rate reveals a distal role for AS160 in insulin-stimulated fusion of GLUT4 storage vesicles. *The Journal of Biological Chemistry*.

[16] Abel E. D., Graveleau C., Betuing S. (2004). Regulation of insulin-responsive aminopeptidase expression and targeting in the insulin-responsive vesicle compartment of glucose transporter isoform 4-deficient cardiomyocytes. *Molecular Endocrinology*.

[17] Subtil A., Lampson M. A., Keller S. R., McGraw T. E. (2000). Characterization of the insulin-regulated endocytic recycling mechanism in 3T3-L1 adipocytes using a novel reporter molecule. *The Journal of Biological Chemistry*.

[18] Lampson M. A., Racz A., Cushman S. W., McGraw T. E. (2000). Demonstration of insulin-responsive trafficking of GLUT4 and vpTR in fibroblasts. *Journal of Cell Science*.

[19] Zhou Q., Yang X., Xiong M. (2016). Chloroquine increases glucose uptake via enhancing GLUT4 translocation and fusion with the plasma membrane in L6 cells. *Cellular Physiology and Biochemistry*.

[20] Wang Q., Khayat Z., Kishi K., Ebina Y., Klip A. (1998). GLUT4 translocation by insulin in intact muscle cells: Detection by a fast and quantitative assay. *FEBS Letters*.

[21] Liu Q.-H., Zheng Y.-M., Korde A. S. (2009). Membrane depolarization causes a direct activation of G protein-coupled receptors leading to local Ca^2+^ release in smooth muscle. *Proceedings of the National Acadamy of Sciences of the United States of America*.

[22] Li Q., Zhu X., Ishikura S. (2014). Ca^2+^ signals promote GLUT4 exocytosis and reduce its endocytosis in muscle cells. *American Journal of Physiology-Endocrinology and Metabolism*.

[23] Whitehead J. P., Molero J. C., Clark S., Martin S., Meneilly G., James D. E. (2001). The Role of Ca^2+^ in Insulin-stimulated Glucose Transport in 3T3-L1 Cells. *The Journal of Biological Chemistry*.

[24] Klip A., Sun Y., Chiu T. T., Foley K. P. (2014). Signal transduction meets vesicle traffic: the software and hardware of GLUT4 translocation. *American Journal of Physiology-Cell Physiology*.

[25] Klip A. (2009). The many ways to regulate glucose transporter 4. *Applied Physiology, Nutrition, and Metabolism*.

[26] Jiang Z. Y., Zhou Q. L., Coleman K. A., Chouinard M., Boese Q., Czech M. P. (2003). Insulin signaling through Akt/protein kinase B analyzed by small interfering RNA-mediated gene silencing. *Proceedings of the National Acadamy of Sciences of the United States of America*.

[27] Martin S. S., Haruta T., Morris A. J., Klippel A., Williams L. T., Olefsk J. M. (1996). Activated phosphatidylinositol 3-kinase is sufficient to mediate actin rearrangement and GLUT4 translocation in 3T3-L1 adipocytes. *The Journal of Biological Chemistry*.

[28] Lee J. O., Lee S. K., Kim J. H. (2012). Metformin regulates glucose transporter 4 (GLUT4) translocation through AMP-activated protein kinase (AMPK)-mediated Cbl/CAP signaling in 3T3-L1 preadipocyte cells. *The Journal of Biological Chemistry*.

[29] Hardie D. G., Sakamoto K. (2006). AMPK: a key sensor of fuel and energy status in skeletal muscle. *Physiology Journal*.

[30] Lee C. T., Ussher J. R., Mohammad A., Lam A., Lopaschuk G. D. (2014). 5′-AMP-activated protein kinase increases glucose uptake independent of GLUT4 translocation in cardiac myocytes. *Canadian Journal of Physiology and Pharmacology*.

[31] Tsuchiya A., Kanno T., Nishizaki T. (2013). Diacylglycerol promotes GLUT4 translocation to the cell surface in a PKCepsilon-dependent and PKClambda/iota and -zeta-independent manner. *Life Sciences*.

[32] Holman G. D., Kozka I. J., Clark A. E. (1990). Cell surface labeling of glucose transporter isoform GLUT4 by bis-mannose photolabel: Correlation with stimulation of glucose transport in rat adipose cells by insulin and phorbol ester. *The Journal of Biological Chemistry*.

[33] Vogt B., Mushack J., Seffer E., Haring H.-U. (1991). The translocation of the glucose transporter sub-types GLUT1 and GLUT4 in isolated fat cells is differently regulated by phorbol esters. *Biochemical Journal*.

